# Supplementary material for: Pulsed Blue Light and Phage Therapy: A Novel Synergistic Bactericide
Source: Antibiotics (Basel). 2025 May 9;14(5):481. doi: 10.3390/antibiotics14050481 (PMC12108478; doi:10.3390/antibiotics14050481)

Supplementary information file

Phage therapy and pulsed blue light: A novel synergistic bactericide

Supplemental Tables

Supplemental Table S1: PBL effect on phage stability
Phages were irradiated with PBL 7.2 mW/cm^2 continuously for 0, 1, 2, 3, or 6h hours. PA14 bacteria in planktonic cultures or in preformed biofilms were incubated overnight with irradiated phage. Twenty-four hours' post-infection, the number of phages (PFU/ml) and the number of bacteria (CFU/ml) were counted. The summary and statistics presented in the table are an average of 5 separate experiments (each an average of triplicate technical repeats):

Treatment	Viral Count [Log PFU/ml]		Planktonic Bacterial Count [Log CFU/ml]		Biofilm Bacterial Count [Log CFU/ml]	
	PASA16	ShipCat1	PASA16	ShipCat1	PASA16	ShipCat1
No Phage	-	-	7.5±0.5	8.6±0.2	7.9±0.1	
Phage	8.5±0.2	8.5±0.1	3.8±0.3*	7.8±0.3*#	5.1±0.5*	6.1±0.3*#
Ph+0.5h (13 J/cm^2)	-	-	-	-	4.8±0.2*	6.1±0.2*#
Ph+1h (26 J/cm^2)	8.4±0.3	8.3±0.3	4±0.3*	7.8±0.3*#	5.1±0.8*	6.2±0.3*#
Ph+1.5h (39 J/cm^2)	-	-	-	-	5.1±0.4*	6.1±0.4*#
Ph+2h (52 J/cm^2)	8.3±0.2	8.4±0.3	3.6±0.2*	7.7±0.2*#	5.1±0.4*	6.0±0.4*#
Ph+3h (78 J/cm^2)	8.4±0.2	8.5±0.2	3.8±0.3*	7.6±0.3*#	-	-
Ph+6h (156 J/cm^2)	8.2±0.3		3.6±0.1*			

Data: mean ± SD; p<0.05 * vs no phage; # vs PASA16; by 2-way ANOVA with Tukey as post hoc.; Note no difference with or without PBL (p>0.5)

Supplemental Table S2: The effect of timing of phage addition
PAShipCat1 phage (10^6 PFU) was added 10, 90, or 150 minutes after treatment with 4.7mW/cm^2 (8.5 J/cm^2) PBL and growth curves (OD_{600}) were measured from which area under the curve at 20 hours was extracted. The summary and statistics presented in the table are an average of 4 separate experiments (each an average of triplicate technical repeats):

**Time of phage addition [min]**	**Control**	**PBL (72%)***	**Phage (49%)*, †**	**P&B (27%)*, #,†**
**10**	28.7±0.9	20.8±0.4 (28%)	15.2±0.3 (47%)	6.1±1.2 (79%)
**90**	28.7±0.9	20.8±0.4 (28%)	15.3±0.4 (47%)	8.3±0.8 (71%)‡
**150**	28.7±0.9	20.8±0.4 (28%)	11.9±0.3 (59%)	9.2±0.6 (68%)‡

Data=mean±SD (%reduction from control); Analysis by 2-way ANOVA with Tukey as post-hoc test: Main effects (group) *, †, # p<0.001 from control, PBL, phage respectively; Interaction within P&B (time of addition) ‡p<0.01 from 10 minutes

Supplemental Table S3: The effect of combinatorial treatment on antibiotic resistance bacteria PATZ2
Antibiotic resistant bacteria PATZ2 were treated with either 2 µg/ml Ceftazidime or with PASA16 10^8 PFU/ml with or without 30 minutes PBL 7.2 mW/cm^2 and growth curves (OD_{600}) were measured from which area under the curve at 20 hours was extracted. The summary and statistics presented in the table are a summary of 5 separate experiments (each an average of triplicate technical repeats):

Group	Control	Ant	PASA16	PBL	PBL&PASA16
Median[IQR]	23.5[6.7]	23.4[7.9]	9.7[1.5]* †	14.8[2.8]* #	3.1[2.3]* †#
Mean±SD	26.7±8	25.8±8.1	9.7±1.2*	15.3±1.8*	3.4±1.8*†

p<0.05 by Kruskal-Wallis with Conovar-Inmann as post hoc or ANOVA with Tukey as post hoc: *from Control or Antibiotics; † from PBL; #from PASA16

Supplemental Table S4: The effect of PBL and phage on biofilm viability
Formed biofilms were irradiated with 26 J/cm2 PBL and live-dead fluorescence was measured 24 hours later. The %Live of total bacteria was determined and the statistics presented in the table are a summary of 5 separate experiments (each an average of triplicate technical repeats):

Group	Control	PASA16	PBL	PBL&PASA16
Median[IQR]	80 [10]	40 [10]*	25 [10]* #	15 [21]* #

*p<0.001 vs control for all; #p<0.05 vs phage alone by Kruskal-Wallis with Conover-Inman as post-hoc

Supplemental Table S5: The effect of PBL and phage on planktonic and biofilm ROS levels
Intracellular ROS levels were assessed using the nitro blue tetrazolium (NBT) assay at 10 minutes, 1 hour, and 3 hours post-treatment. Irradiation protocol and phage concentration as described above for planktonic and biofilm assays. The summary and statistics presented in the table are an average of 5 separate experiments (each an average of triplicate technical repeats)

System	Time	10 minute	1h	3h
Planktonic	PBL&CTR	1.36±0.27	0.96±0.05	1.04±0.03
	PBL&PASA	1.69±0.13*#	0.88±0.10	0.99±0.06
	SHAM&PASA	0.96±0.17	0.89±0.24	0.92±0.10
Biofilm	PBL&CTR	1.02±0.17	1.09±0.24	1.06±0.22
	PBL&PASA	0.93±0.05	0.65±0.14*	1.54±0.23*†#
	SHAM&PASA	1.10±0.09	0.90±0.19	1.15±0.10

p<0.05 *vs control, †vs PBL, #vs PASA by ANOVA+Tukey

Supplemental Figure S1

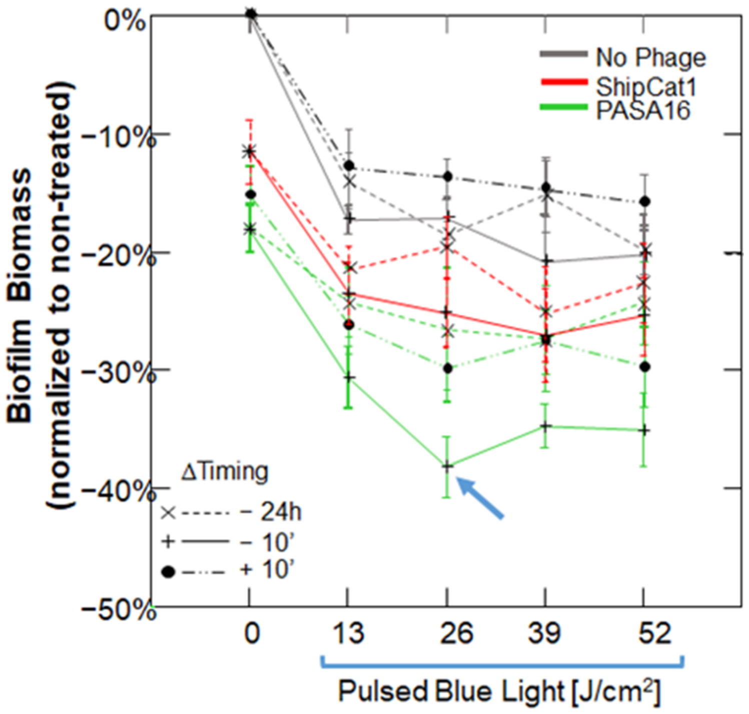

Supplement: Supplementary file 1 [file antibiotics-14-00481-s001.zip › antibiotics-3529408-supplementary.pdf]
